# Water vapor sorption properties of cellulose nanocrystals and nanofibers using dynamic vapor sorption apparatus

**DOI:** 10.1038/s41598-017-14664-7

**Published:** 2017-10-27

**Authors:** Xin Guo, Yiqiang Wu, Xinfeng Xie

**Affiliations:** 1 College of Material Science and Engineering, Central South University of Forestry and Technology, Changsha, 410004 China; 2 School of Forest Resources and Environmental Science, Michigan Technological University, Michigan, 49931 United States

## Abstract

Hygroscopic behavior is an inherent characteristic of nanocellulose which strongly affects its applications. In this study, the water vapor sorption behavior of four nanocellulose samples, such as cellulose nanocrystals and nanofibers with cellulose I and II structures (cellulose nanocrystals (CNC) I, CNC II, cellulose nanofibers (CNF) I, and CNF II) were studied by dynamic vapor sorption. The highly reproducible data including the running time, real-time sample mass, target relative humidity (RH), actual RH, and isotherm temperature were recorded during the sorption process. In analyzing these data, significant differences in the total running time, equilibrium moisture content, sorption hysteresis and sorption kinetics between these four nanocellulose samples were confirmed. It was important to note that CNC I, CNC II, CNF I, and CNF II had equilibrium moisture contents of 21.4, 28.6, 33.2, and 38.9%, respectively, at a RH of 95%. Then, the sorption kinetics behavior was accurately described by using the parallel exponential kinetics (PEK) model. Furthermore, the Kelvin-Voigt model was introduced to interpret the PEK behavior and calculate the modulus of these four nanocellulose samples.

## Introduction

Nanocellulose has excellent physical, chemical, and biological properties, such as good thermal stability, high strength, low degradation, and nontoxicity^1,2^. Nanocellulose can be derived from agricultural residue, such as coconut husk fibers, cassava bagasse, banana rachis, mulberry bark, soybean pods, wheat, straw, and soy hulls, with low preparation cost^3–6^; it has been widely used in automobile parts and may find application in tissue engineering and green packaging materials^7^. Therefore, the potential use of nanocellulose has been gaining popularity in many scientific fields, such as materials science, electronics, and biomedicine. However, hydrophilic nanocellulose adsorbs water under hygrothermal conditions, which strongly affects its surface behavior and leads to reliability problems^8,9^. Consequently, the water vapor sorption behavior of nanocellulose needs to be understood prior to the commercial utilization of nanocellulose.

Nanocellulose refers to rod-like cellulose nanocrystals and needlelike cellulose nanofibers whose structures differ from the normal cellulose. It has been proven that the structure and composition are important factor for controlling the water vapor sorption behavior^10^. Therefore, the water vapor sorption behavior of nanocellulose is complex and different to that of the normal cellulose materials. There are some representative publications available which have provided lots of useful information about the interactions between nanocellulose and water^11^. However, due to the complexity of water vapor sorption behavior, there are still many issues that deserve more detailed investigation, especially in the case of cellulose nanocrystals and cellulose nanofibers.

Water vapor sorption behaviour of cellulose has been studied by a number of experimental methods^12–16^. Among these methods, dynamic vapor sorption (DVS) method offers distinct advantages because of the highly reproducible data over a wide relative humidity (RH) range in real time^11,17,18^. Using this method, the sorption isotherms of cellulose materials as well as the extent of sorption hysteresis have been obtained^19^. The water vapor sorption behavior of many cellulose materials, including natural fibers^20,21^, regenerated cellulose^22^, microcrystalline cellulosic fibers^23^, and wood powder^24^, have been analyzed by using the parallel exponential kinetics (PEK) model as follows:

1 $$MC=M{C}_{0}+M{C}_{1}(1-\exp (-t/{t}_{1}))+M{C}_{2}(1-\exp (-t/{t}_{2}))$$

Where, *MC* is moisture content, *MC*_0_ is moisture content at the initial time, *MC*_1_ is moisture content related to the fast regime, *MC*_2_ is moisture content related to slow regime, *t*_1_ is the time to reach equilibrium in the fast regime, and *t*_2_ is the time to reach equilibrium in the slow regime. Clearly, in the PEK model, the water vapor sorption behavior consists of a fast and slow regime. The fast regime is assigned to the inflow of water onto the surface. The slow regime is related to new sorption points^22,25–27^. Both the regimes can be described by the Kelvin-Vogit model as follows:

2 $$\varepsilon =({\sigma }_{0}/E)[{\rm{1}}-\exp (-\lambda t)]$$

Where, ε is strain, *σ*_0_ is the applied stress, *E* is the elastic modulus, *λ* is a constant equal to *E*/*η*, and *η* is viscosity. In the analysis of the PEK behavior of cellulose material, the strain is considered to be volume change of the cellulose material caused by the water vapor sorption. Meanwhile, the applied stress is swelling pressure caused by the existence of water in the cellulose material leading to a relevant volume change. The swelling pressure can be calculated using the following formula.

3 $${\sigma }_{0}=-(\rho /M)RT\cdot \,\mathrm{ln}({p}_{i}/{p}_{f})$$

Where, *ρ* and *M* are density and molecular weight of water, *R* is gas constant, *T* is Kelvin temperature, and *p*_*i*_ and *p*_*f*_ are the initial and final water vapor pressure. The PEK and Kelvin-Voigt models can be combined to analyze the water vapor sorption kinetics^28,29^.

In this work, we used the DVS technique to study the water vapor sorption behavior of four nanocellulose samples (i.e., CNC I, CNC II, CNF I, and CNF II). The objective of this study was to examine the effects of nanocellulose types caused by different preparation procedures on the total running time, equilibrium moisture content, sorption hysteresis, and sorption kinetics.

## Results

### Water vapor sorption behavior

**Figure 1:**
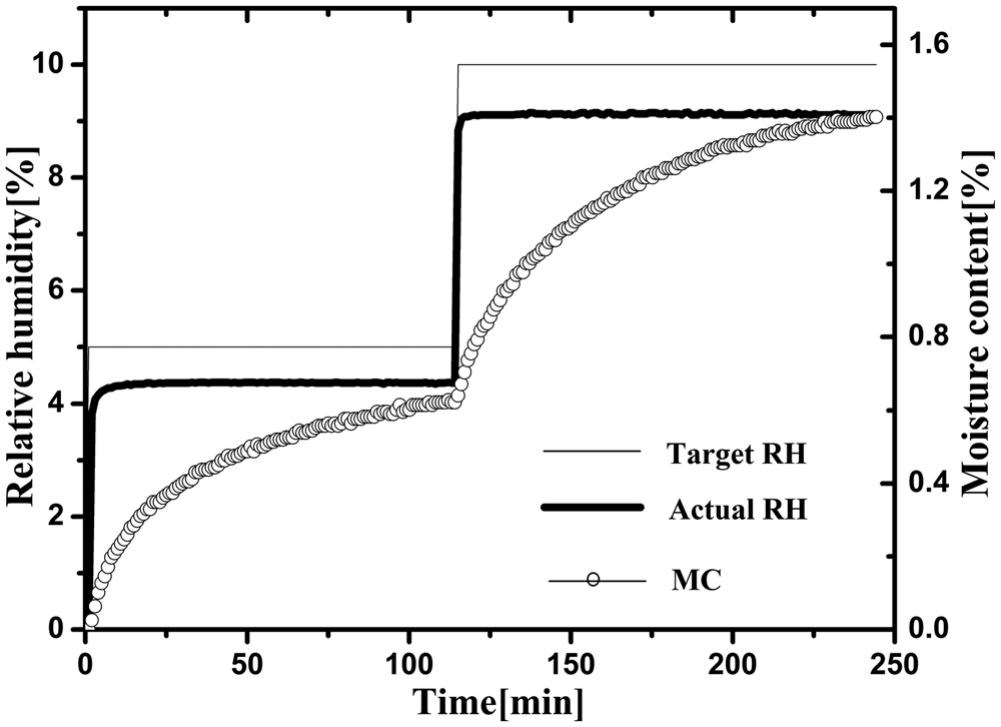
Water vapor adsorption behavior of CNC I at the RH of 5 and 10% indicating target RH (narrow line), actual RH (bold line), and moisture content (circle points).

**Figure 2:**
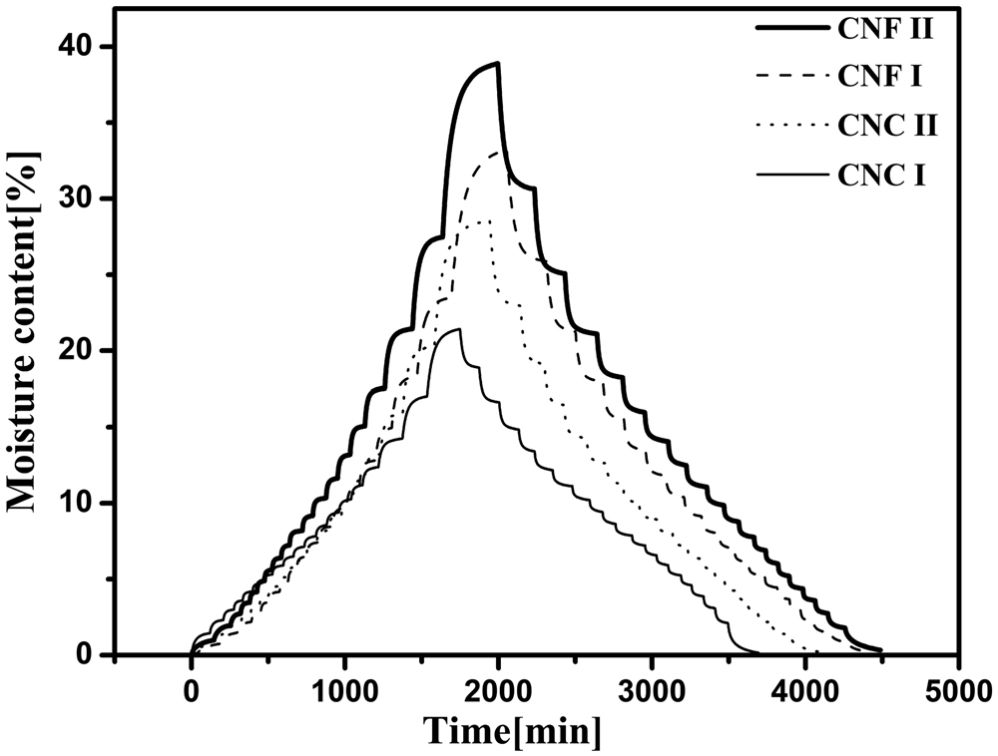
Moisture content of four nanocellulose samples during the sorption process.

The total running time is an important parameter for characterizing the water vapor sorption behavior of the nanocellulose, which includes the time taken during the adsorption cycle and that required for the subsequent desorption cycle. As shown in Fig. 1, after changing the target RH to a new setting, there was typically a delay of approximately 4–10 min in which the actual RH approached the set target RH. After the initial few minutes, the actual RH was kept stable, and then the fluctuation of the actual RH value was less than 0.1% at extended time. Meanwhile, after changing the target RH, the real-time moisture content of the sample generated an asymptotic curve against time, and it could reach the equilibrium moisture content (EMC) if the stability time was long enough. Once the change of moisture content was less than 0.002% min^−1^ after 10 min, the target RH was changed to the next setting. Over the time profile in the isotherm run, the total running times for the four nanocellulose samples are shown in Fig. 2. Although the values were very close, the total running times for four nanocellulose samples increased in the following order: CNC I < CNC II < CNF I < CNF II. The total running time is related to the water adsorption so that it increases with increase in the water adsorption. As shown in Fig. 2, the total running times of CNC were less than those of CNF. This is likely due to the lower sorption ability of CNC compared to that of CNF. The relatively high sorption ability of CNF could be due to its more amorphous nature. The total running times of the nanocellulose with cellulose I structure were less than those of the nanocellulose with cellulose II structure throughout the entire RH range. The reason probably is that the swelling happens more in the case of the nanocellulose with cellulose II structure. In addition, as shown in Fig. 2, the total running time for the desorption process was slightly longer than that for the adsorption process which is another way to observe the phenomena behind the hysteresis.

**Figure 3:**
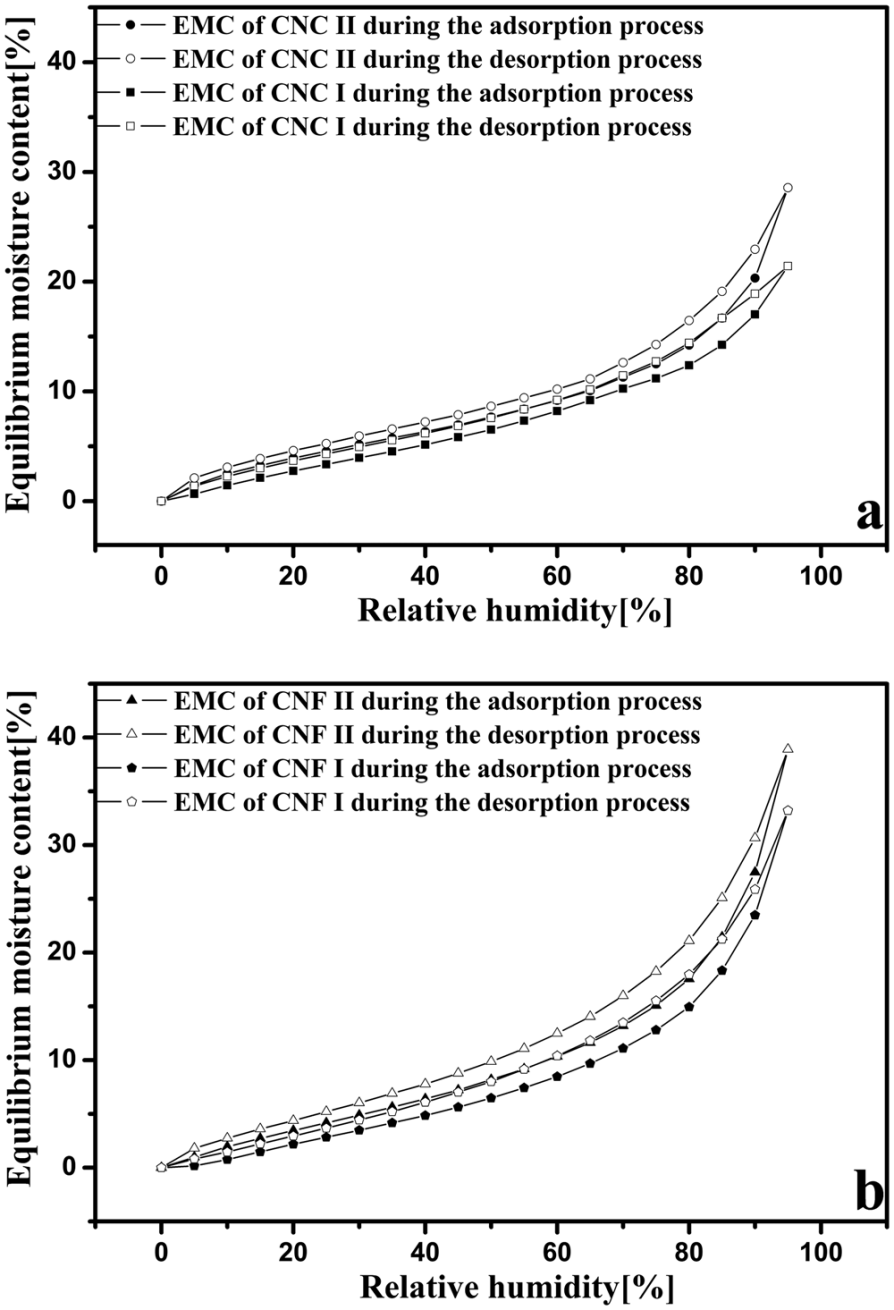
Equilibrium moisture content of CNC I (**a**), CNC II (**a**), CNF I (**b**), and CNF II (**b**) during the adsorption (solid points) and desorption (empty points) processes.

Equilibrium moisture content (EMC) is another important parameter for characterizing water vapor sorption. As expected, the EMC of these four cellulose samples, as a function of RH, generated typical sigmoidal isotherms in both the adsorption and desorption processes, as shown in Fig. 3. Similar EMC isotherms were observed in other normal cellulose materials^23^. As shown in Fig. 3, the EMC increased rapidly when the RH was above 80%. The reason for this rapid increase could be capillary condensation, a phenomenon already observed in the analysis of crystalline cellulose^30^. These four cellulose samples at 95% RH had EMC values of 21.4, 28.6, 33.2, and 38.9%, respectively. These values were higher than those of the other cellulose fibers, such as cotton linter and α-cellulose^31^. This was attributed to the larger pore volume and pore diameter of the CNC and CNF films that were made of nanoparticles^32^. The EMC of CNF was higher than that of the CNC owing to the lower degree of crystallinity and more amorphous nature of CNF. In addition, the EMCs of cellulose nanocrystals and nanofibers with cellulose II structures were higher than those with cellulose I structure. These differences are derived from crystalline structure of cellulose. During the preparation of CNC II and CNF II, mercerization treatment (20 wt% NaOH) was carried out for the raw material (bleached wood pulp), which modified the crystalline structure from I to II. It is generally known that the mercerization increases the amount of amorphous cellulose at the expense of crystalline cellulose. Meanwhile, the important modification of mercerization is removal of hydrogen bonding in the network structure. Therefore, the cellulose nanocrystals and nanofibers with cellulose II structure have higher water sorption capacity than those with cellulose I structure^33^. Moreover, Han *et al*. used Fourier Transform Infrared (FTIR) spectroscopy to confirm this conclusion^34^.

### Sorption hysteresis

**Figure 4:**
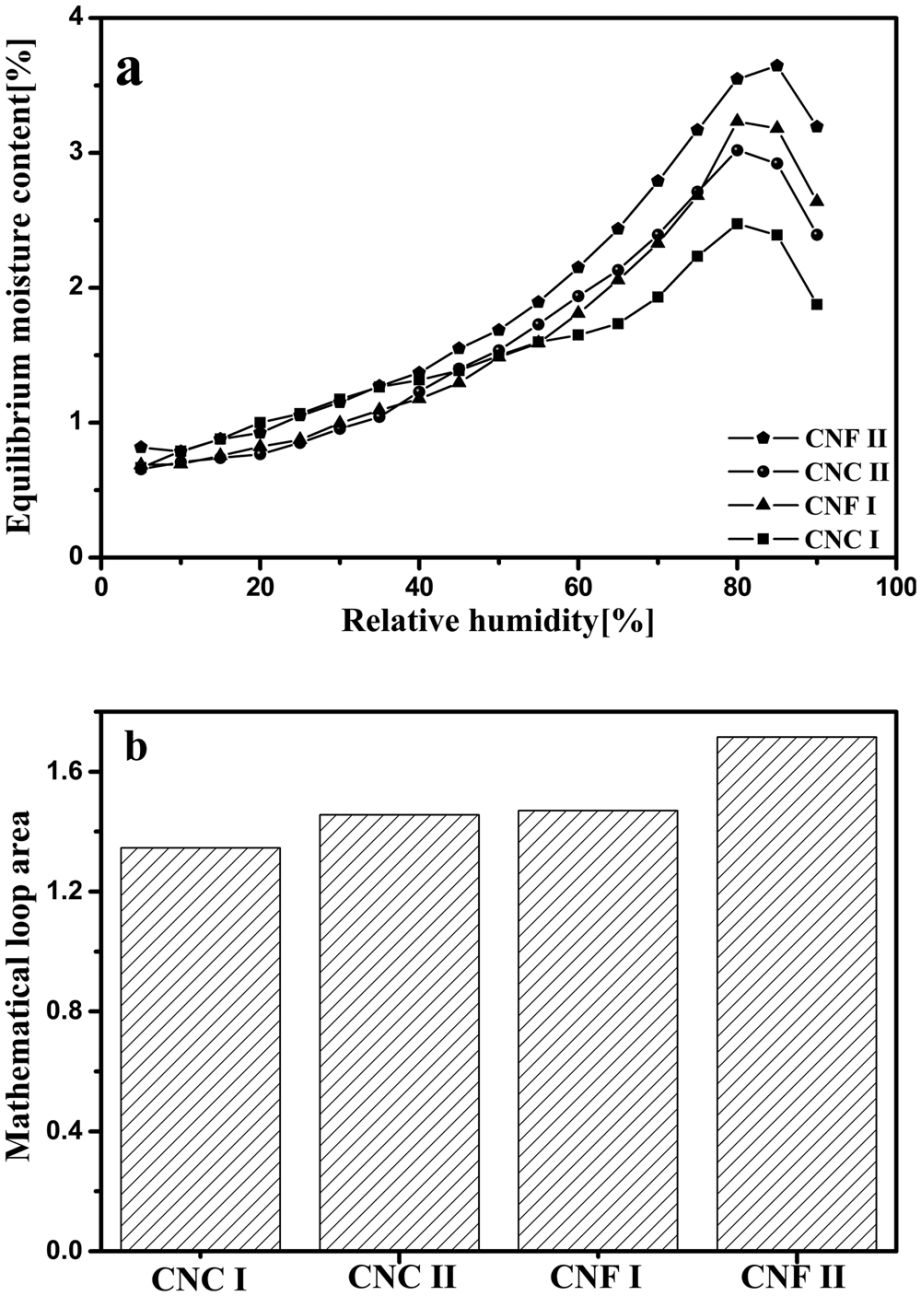
Sorption hysteresis (**a**) and mathematical loop area (**b**) of four nanocellulose samples.

Figure 4 shows sorption hysteresis of these four nanocellulose samples over the full RH range. High sorption hysteresis was clearly observed in the high RH region. It has been considered that the hysteresis phenomenon of cellulosic materials is resulted from the response delay caused by the collapse of nanopores in the interfibrillar matrix as the internal water molecules exit as well as the delay of structural deformation during the adsorption process. To explore the total sorption hysteresis, the mathematical areas of the isotherm loops were calculated (Fig. 4b). Similar methods have been reported by Xie *et al*.^26^. The mathematical areas of the isotherm loops for CNF were larger than those for CNC, which confirmed that the total sorption hysteresis of CNF were greater than those of CNC. Meanwhile, compared with the nanocellulose with cellulose I structure, the nanocellulose with cellulose II structure had greater total sorption hysteresis. The variations in the total sorption hysteresis were attributed to structural swelling^35^. As mentioned earlier in this paper, the amorphous content of CNF is more than that of CNC. Accordingly, the extent of structural swelling of CNF would be greater than with CNC. In addition, the amorphous contents of cellulose nanocrystals and nanofibers with cellulose II structure were more than those with cellulose I structure. So the extents of structural swelling of cellulose nanocrystals and nanofibers with cellulose II structure would be greater than those with cellulose I structure. All these may account for the aforementioned variations in the total sorption hysteresis.

**Figure 5:**
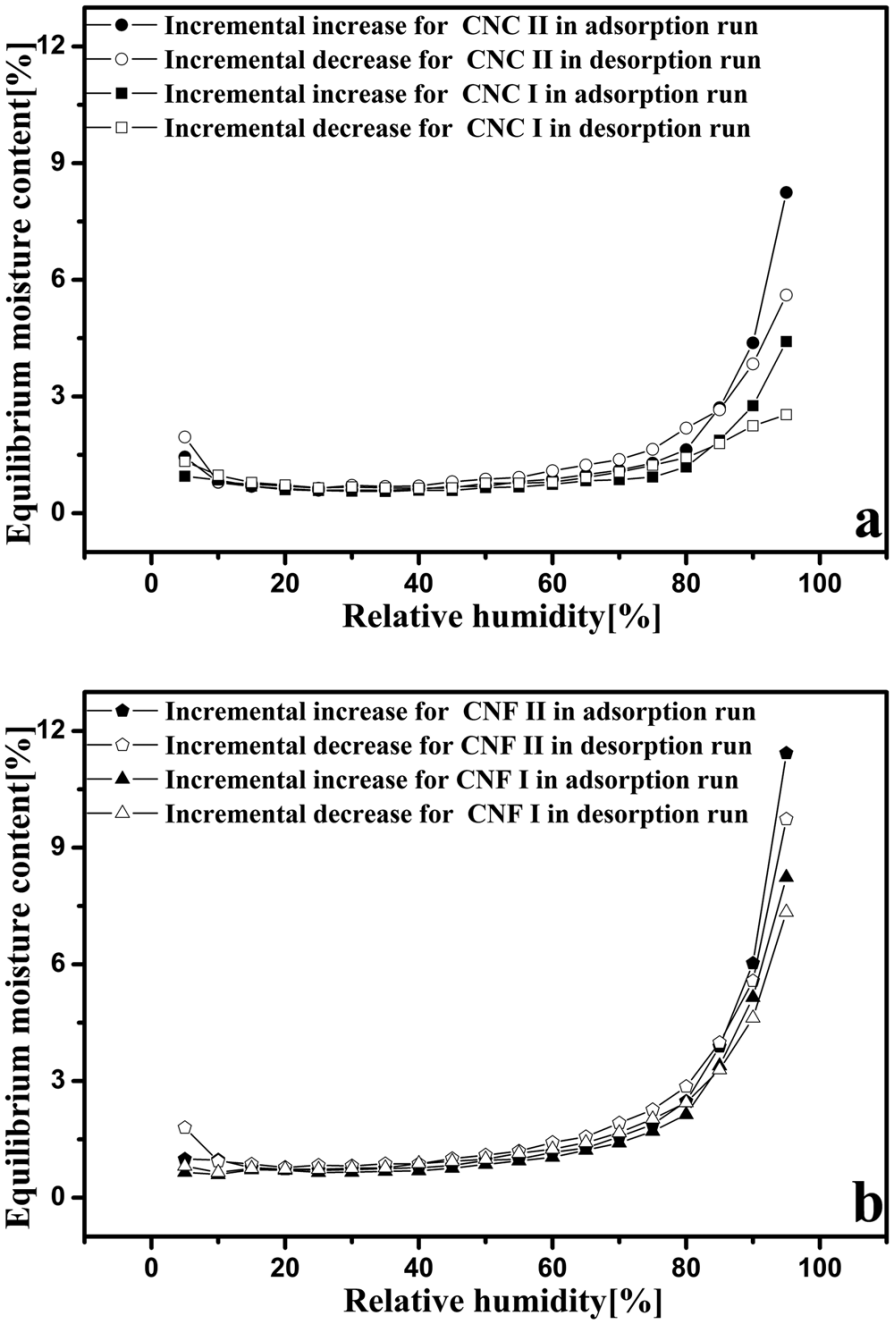
Incremental increases and decreases in EMC for CNC I (**a**), CNC II (**a**), CNF I (**b**), and CNF II (**b**) in the adsorption (solid points) and desorption (empty points) runs.

To further show the hysteresis, the incremental increases and decreases in EMC vs. RH are shown in Fig. 5. At the upper end of the RH range, the incremental increase in EMC during adsorption and the incremental decrease in EMC during desorption were observed clearly. However, in the other regions of the RH range, the incremental increases and decreases in EMC were not so obvious. Among these four nanocellulose samples, CNF II showed the most obvious incremental increases and decreases in EMC (Fig. 5b). Meanwhile, the incremental increases and decreases in EMC for the nanocellulose with the cellulose II structure were greater than those for the nanocellulose with the cellulose I structure. All these differences are attributed to the differences in the crystalline structure.

### Sorption kinetics

**Figure 6:**
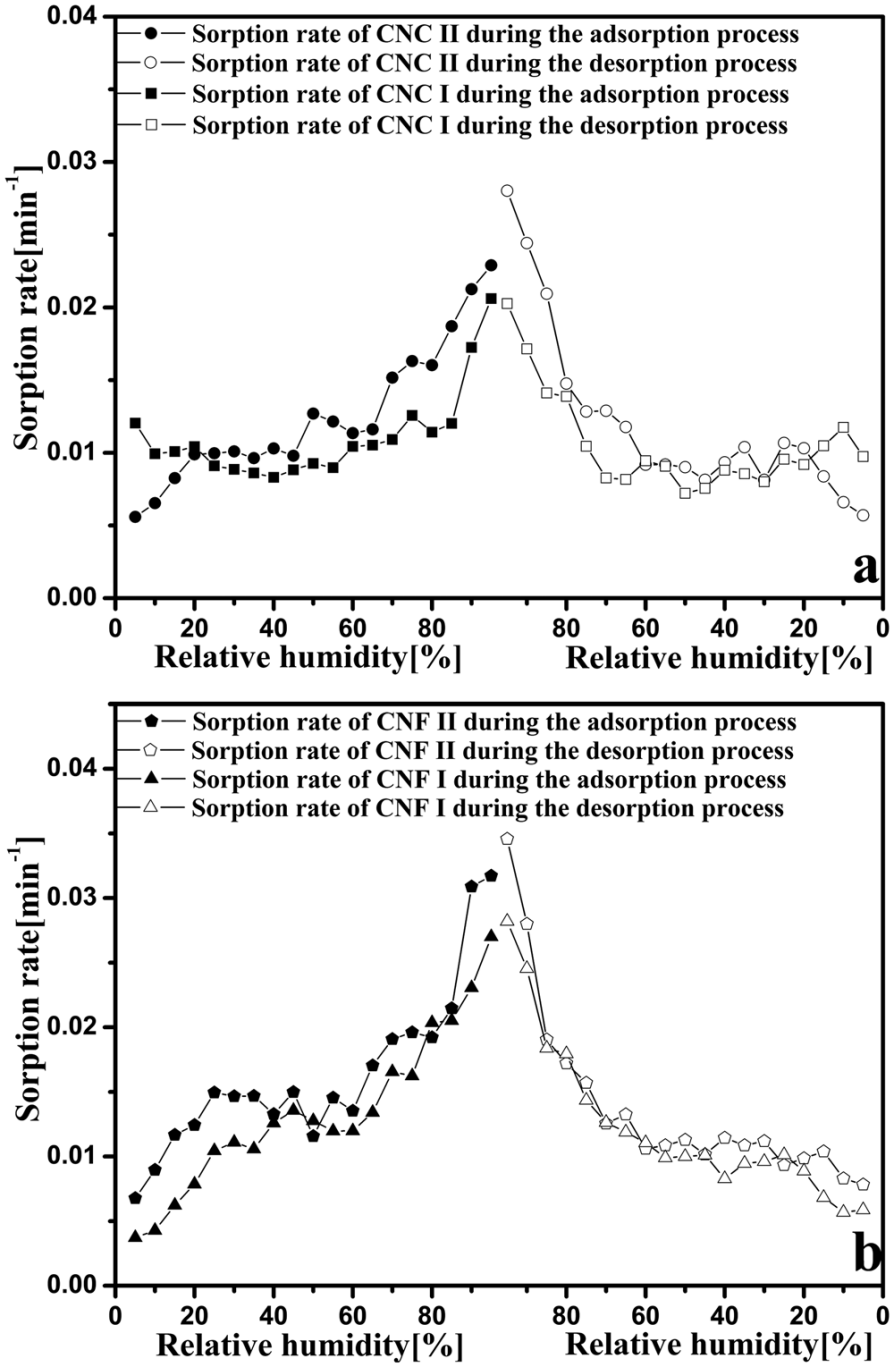
Average sorption rates of CNC I (**a**), CNC II (**a**), CNF I (**b**), and CNF II (**b**) during the adsorption (solid points) and desorption (empty points) processes.

Figure 6 shows the average rate of sorption at various RHs during adsorption and desorption, in which the average rate at the specific RH was acquired by dividing the increment or decrement of EMC by time. The shape of the measured average rates for all four nanocellulose samples was an inverted V. This suggests that the average sorption rate is high at high RHs; while, in the low RH range during sorption and desorption, the average sorption rate is small.

**Figure 7:**
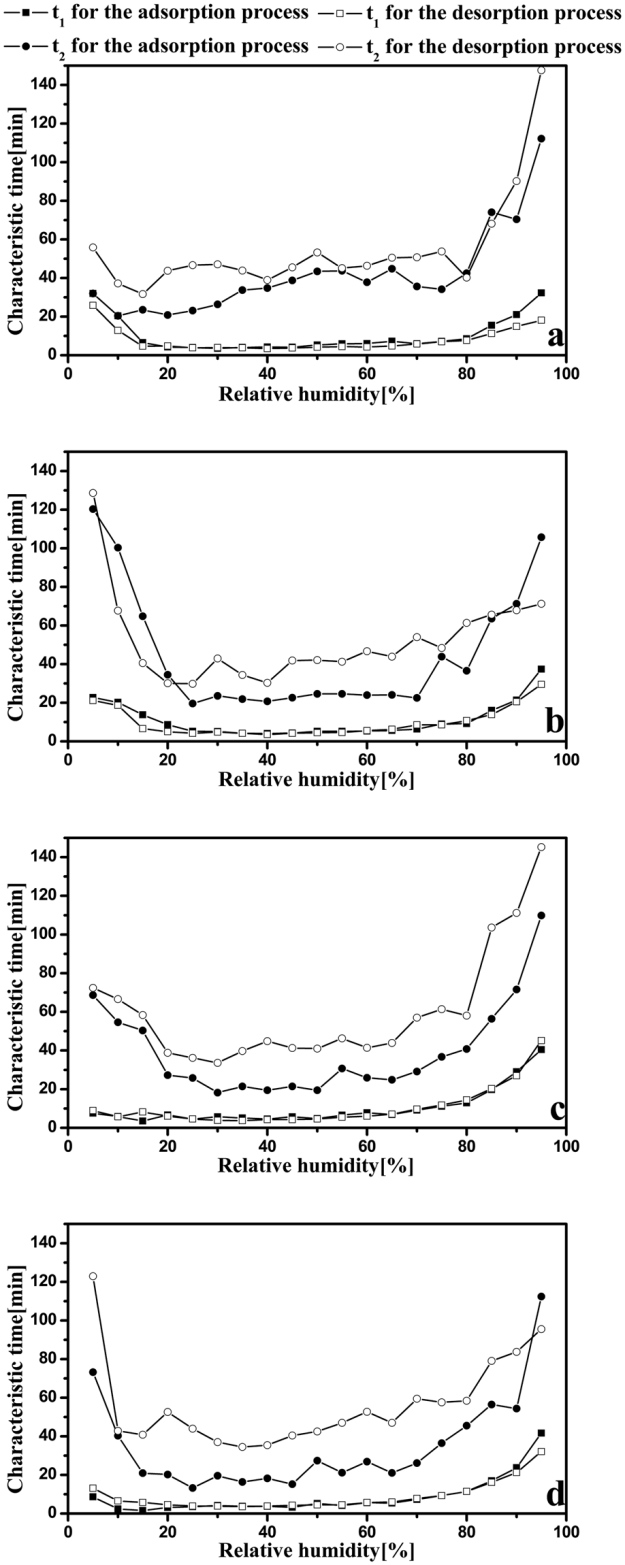
Characteristic times *t*_1_ and *t*_2_ of CNC I (**a**), CNC II (**b**), CNF I (**c**), and CNF II (**d**) for the adsorption (solid points) and desorption (empty points) processes.

**Figure 8:**
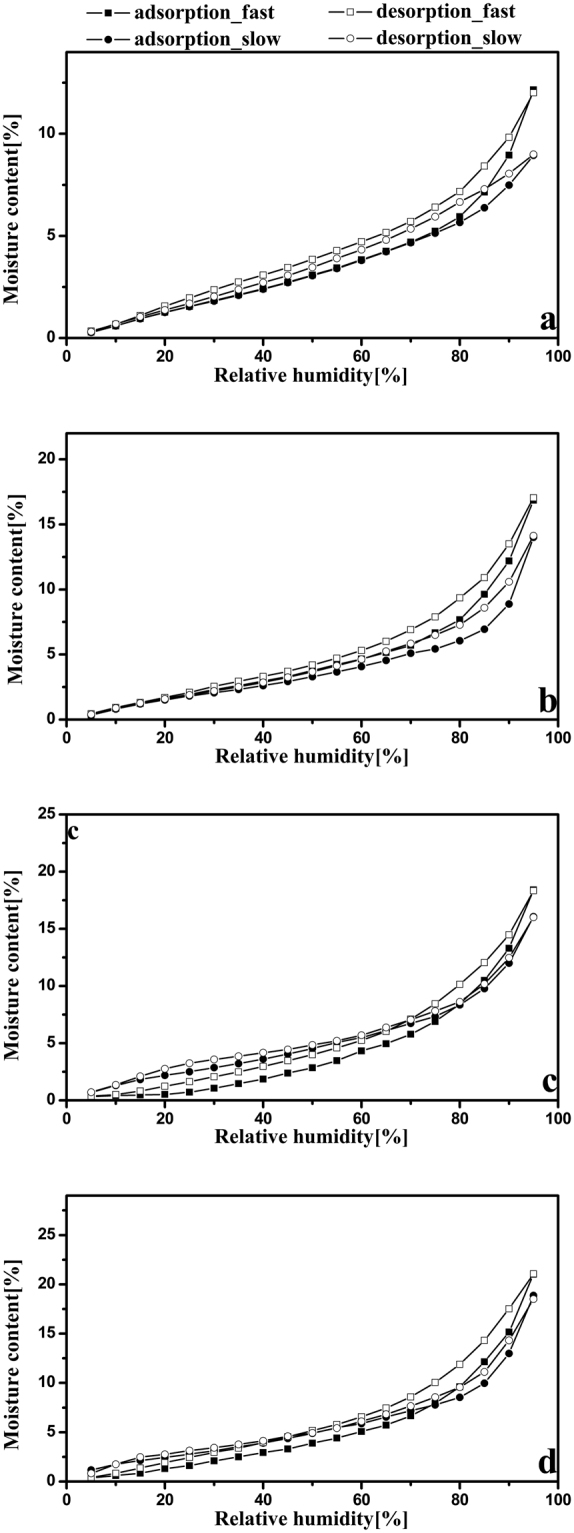
Cumulative moisture contents *MC*_1_ and *MC*_2_ of CNC I (**a**), CNC II (**b**), CNF I (**c**), and CNF II (**d**) for the adsorption (solid points) and desorption (empty points) processes.

The experimental data of dynamic moisture content at each RH level was curve-fitted by using the PEK model and the PEK parameters of *t*_1_ and *t*_2_ are shown in Fig. 7. Time *t*_1_ and *t*_2_ varied during adsorption and desorption process, especially at high and low RH; this variation in time *t*_2_ was also observed by Xie *et al*. in microcrystalline cellulose^31^. There was an obvious numerical difference between the adsorption and desorption characteristic times with the slow kinetic processes (*t*_2_). However, the numerical difference between the adsorption and desorption characteristic times with the fast kinetic processes was not so clear (*t*_1_). All these suggest that the adsorption and desorption are asymmetric processes. Cumulative moisture content related to the fast and slow regimes in the sorption process are shown in Fig. 8. Both CNC I and CNC II gained more mass during the fast regime than the slow regime (Fig. 8a,b), and it can be concluded that the fast regime was the dominant regime in the full RH range. The same conclusion has been evident in other measurements of cotton linter and microcrystalline cellulose^31^. However, for CNF I and CNF II, there were more complex variations in the cumulative *MC*_1_ and *MC*_2_ (Fig. 8c,d). Remarkably, the crossovers of cumulative *MC*_1_ and *MC*_2_ showed the changes of CNF I and CNF II in PEK behavior with RH. So far, there is no reasonable explanation for these changes. In addition, at the highest RH of 95%, the cumulative MC of both the fast and slow regimes increased in the order of CNC I < CNC II < CNF I < CNF II (Fig. 8). As mentioned above, the fast regime is related to readily accessible sorption sites on the surface, whereas the slow regime is linked to the production of new water adsorption sites. Therefore, the increase of the cumulative MC associated with the fast regime may be due to the increase of hydroxyl groups whose growth order is CNC I < CNC II < CNF I < CNF II confirmed by Han *et al*.^34^. Meanwhile, the cumulative MC associated with the slow regime in this study may be mainly attributed to the structural swelling opens up new sorption sites. The extents of structural swelling of these four nanocellulose samples were proved to increase in the same order.

### The applicability of Kelvin-Voigt model

**Figure 9:**
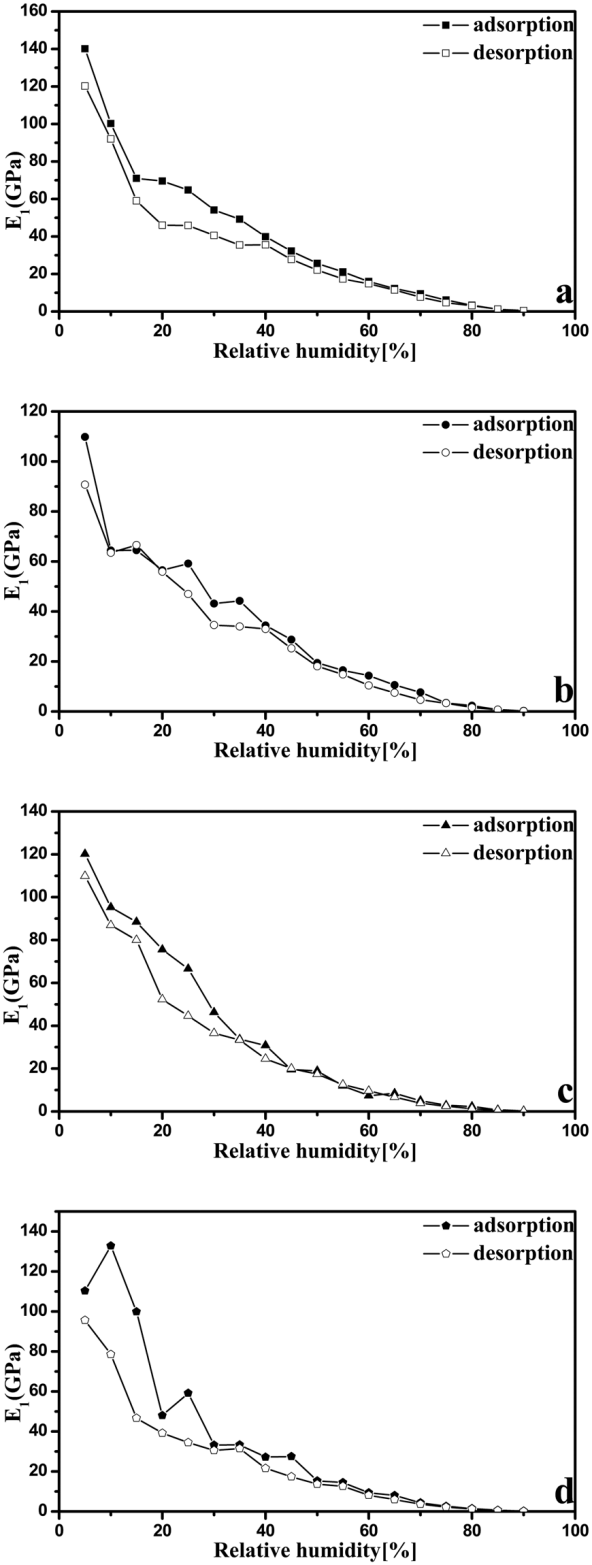
*E*_1_ of CNC I (**a**), CNC II (**b**), CNF I (**c**), and CNF II (**d**) in the adsorption (solid points) and desorption (empty points) processes.

**Figure 10:**
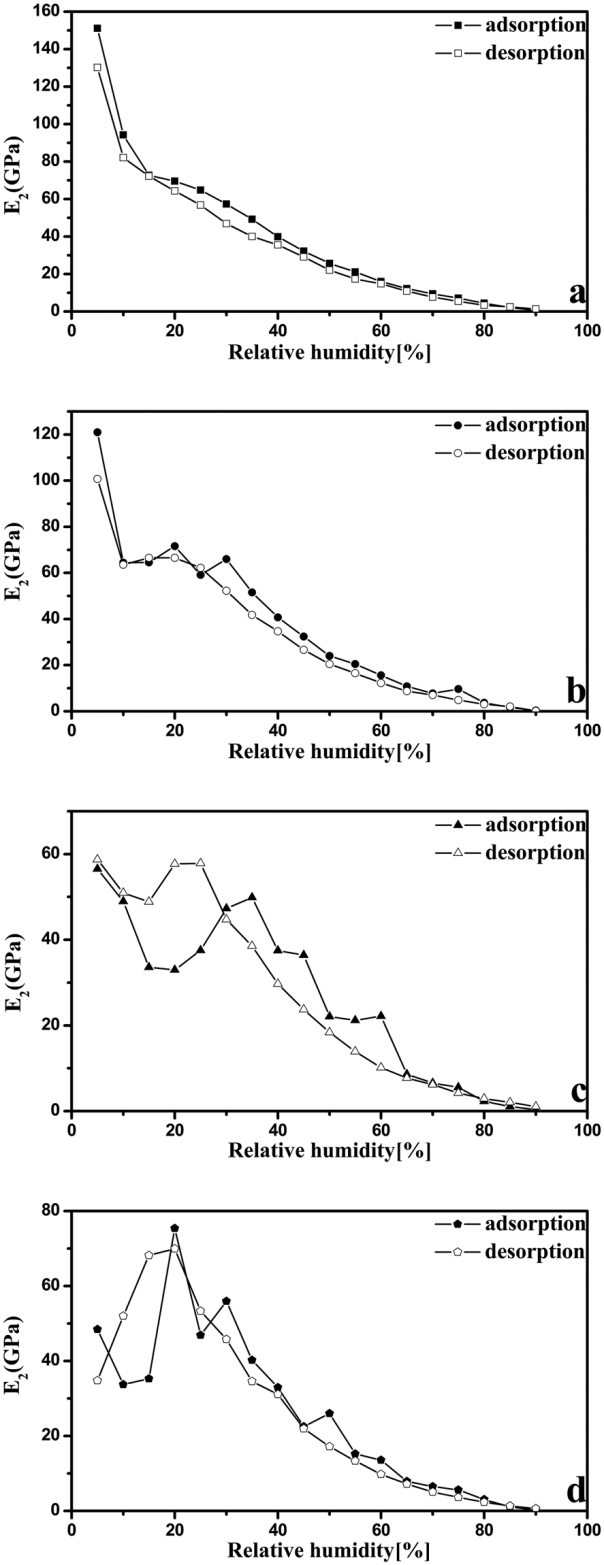
*E*_2_ of CNC I (**a**), CNC II (**b**), CNF I (**c**), and CNF II (**d**) in the adsorption (solid points) and desorption (empty points) processes.

The Kelvin-Voigt model was introduced to estimate the modulus of four nanocellulose samples; similar calculation methods have been previously reported in the literature^31^. As shown previously, the kinetics of adsorption or desorption consist of a fast and a slow regime, with separate moduli^36,37^. In this study, the subscript in the modulus (*E*) was used to distinguish between the fast and slow regime (subscript 1 for the fast and subscript 2 for the slow). The moduli of the fast and slow regimes for these four nanocellulose samples are shown in Figs 9,10. As expected, the values of elastic modulus decrease with increasing RH, which is consistent with the effect of absorbed water on the plasticization of nanocellulose. There is no previous report of the modulus of CNC and CNF calculated using Kelvin-Vogit model. However, our group has determined the modulus of CNC and CNF films using Instron 5582 testing machine and ASTM D638-10 standard^38–40^. Here, we used the same procedures to determine the modulus values of CNC I, CNC II, CNF I, and CNF II which were 6.3 ± 0.3, 5.3 ± 0.2, 5.0 ± 0.2, and 3.8 ± 0.2 GPa. It is clearly that the modulus values of 23.9 ± 1.8, 20.5 ± 2.4, 19.2 ± 1.8, and 18.0 ± 4.4 GPa (at 50% RH) calculated using Kelvin-Vogit model in this study for CNC I, CNC II, CNF I, and CNF II are higher than those determined by tension method. Meanwhile, the modulus determined using tension method are acquired by applying external pressure on the nanocellulose, whereas the modulus calculated using Kelvin-Vogit model are based on an internal stress (the swelling pressure). Although the testing mechanism is different, the obtained modulus values are of the same order of magnitude. In addition, previous research has reported that the CNC film has modulus values ranging between 3 and 25 GPa and another studies indicate the CNF film has modulus values ranging between 5 and 21 GPa^11,41–44^. It is important to note that the modulus values calculated using Kelvin-Vogit model are in the range of modulus values quoted in the literature.

## Conclusions

By using DVS technique, a detailed investigation on the water vapor sorption behavior of four nanocellulose samples (i.e., CNC I, CNC II, CNF I, and CNF II) was achieved during the sorption process. The obtained data concerning the running time, real-time sample mass, target RH, and actual RH provided convenient condition for detailed analysis of differences in the total running time, equilibrium moisture content, sorption hysteresis and sorption kinetics between these four nanocellulose samples. It was noteworthy that these four cellulose samples at 95% RH had EMC values of 21.4, 28.6, 33.2, and 38.9%, respectively. Meanwhile, the sorption kinetics behavior was accurately described by using the parallel exponential kinetics (PEK) model which divided the sorption kinetics curve into fast and slow regimes. Furthermore, two Kelvin-Voigt elements were introduced to interpret the PEK behavior and calculate the modulus of these four nanocellulose samples. It is important to note that these four cellulose samples has modulus values of 23.9 ± 1.8, 20.5 ± 2.4, 19.2 ± 1.8, and 18.0 ± 4.4 GPa, and these calculated using Kelvin-Vogit model in this study are of the same order of magnitude as values quoted in the literature.

## Methods

### Nanocellulose materials

This work was focused on four typical nanocellulose samples: CNC I, CNC II, CNF I, and CNF II. The preparation procedures for these four nanocellulose samples were clearly reported by Han *et al*.^34^; therefore only a brief description of their preparation is provided here. For CNC I, dried bleached wood pulp (W-50 grade of KC Flock, Nippon Paper Chemicals Co., Tokyo, Japan) was raw material and it was hydrolyzed for 1 h with 64 wt % sulfuric acid and then was filtered under vacuum. The filtered material was mixed with distilled water thoroughly for 20 min and then centrifuged at 26 °C for three cycles. The suspension was obtained by centrifuging after each washing and dialyzed against distilled water in regenerated cellulose dialysis tubes with a molecular weight cutoff of 12000–14000 (Fisher Scientific, Pittsburgh, PA, U.S.A.). This process was repeated for several days until a pH neutral solution was obtained. This material was then put into a high-pressure homogenizer (Microfluidizer M-110P, Microfluidics Corp., Newton, MA, U.S.A.) at a rate of 135 mL/min for five times. After homogenization, the concentration of CNC I suspension was 1.0 wt % and was adjusted to 0.1 wt % by adding water. Meanwhile, for preparing the CNF I, the concentration of sulfuric acid was changed to 48 wt %, while the other conditions were kept unchanged. In addition, for preparing the CNC II and CNF II, bleached wood pulp was pretreated. Dry bleached wood pulp was mercerized using 20 wt % NaOH solutions for 3 h, and then was filtered and washed by distilled water until a pH neutral solution was obtained. This mercerized bleached wood pulp was the raw material for the production of CNC II and CNF II, and the preparation procedures were the same as those for the CNC I and CNF I. The obtained suspensions of these four nanocellulose samples were then quickly frozen at −75 °C for about 2 h and freeze-dried to form films. The final film was placed between two cover slips and then was sealed in plastic bags.

### DVS setup

A DVS apparatus (DVS AdvantagePlus, Surface Measurement Systems Ltd, London, United Kingdom) was used to determine the isotherms and dynamic sorption behavior of these four nanocellulose samples. The sample masses of CNC I, CNC II, CNF I and CNF II were 13.2, 13.4, 13.3, and 13.2 mg, separately. The data such as the running time, real-time mass of the samples, and the actual RH at a constant temperature of 25 °C were obtained during the sorption process. The RH was set to change from 0% to 95% in 5% steps and then decrease to 0%. At every stage, the RH was kept constant for some time and then increased to the next stage as the changes in sample mass were smaller than 0.002% per minute. Remarkably, the sorption isotherms of nanocellulose sample were recorded as least three times in the preliminary study. Based on these, the DVS apparatus was confirmed to be able to give reproducible sorption data for the nanocellulose and one test was acceptable.

### Data availability statement

The data that support the findings of this study are available from the corresponding author upon reasonable request.
